# Preliminary Experience With Low Molecular Weight Heparin Strategy in COVID-19 Patients

**DOI:** 10.3389/fphar.2020.01124

**Published:** 2020-08-06

**Authors:** Pasquale Paolisso, Luca Bergamaschi, Emanuela Concetta D’Angelo, Francesco Donati, Maddalena Giannella, Sara Tedeschi, Renato Pascale, Michele Bartoletti, Giulia Tesini, Mauro Biffi, Benilde Cosmi, Carmine Pizzi, Pierluigi Viale, Nazzareno Galié

**Affiliations:** ^1^Unit of Cardiology, Department of Experimental, Diagnostic and Specialty Medicine-DIMES, University of Bologna, Bologna, Italy; ^2^Unit of Infectious Diseases, Department of Medical and Surgical Sciences, S. Orsola Hospital, University of Bologna, Bologna, Italy; ^3^Unit of Angiology & Blood Coagulation, S. Orsola-Malpighi University Hospital, Bologna, Italy

**Keywords:** COVID- 19, heparin, LMWH (low molecular weight heparin), in-hospital mortality, DVT prophylaxis

## Abstract

**Background:**

Heparin administration in COVID-19 patients is recommended by expert consensus, although evidence about dosage, duration and efficacy are limited. We aim to investigate the association between different dosages of low molecular weight heparin (LMWH) and mortality among COVID-19 hospitalized patients.

**Methods and Results:**

Retrospective study of 450 laboratory-confirmed COVID-19 patients admitted to Sant’Orsola Bologna Hospital from March 01 to April 10, 2020. Clinical, laboratory and treatment data were collected and analyzed. The in-hospital mortality between COVID-19 patients treated with standard prophylactic LMWH dosage vs. intermediate LMWH dosage was compared. Out of 450 patients, 361 received standard deep vein thrombosis (DVT) prophylaxis enoxaparin treatment (40-60mg daily) and 89 patients received intermediate enoxaparin dosage (40–60 mg twice daily) for 7 days. No significant differences in the main demographic characteristics and laboratory testings at admission were observed in the two heparin regimen subgroups, except for older age and prevalence of hypertension in the group treated with “standard” prophylaxis LMWH dosage. The intermediate LMWH administration was associated with a lower in-hospital all-cause mortality compared to the “standard” prophylactic LMWH dosage (18.8% vs. 5.8%, p = 0.02). This difference remained significant after adjustment with the propensity score for variables that differed significantly between the dosage groups (OR= 0.260, 95% CI 0.089–0.758, p=0.014).

**Conclusions:**

Intermediate LMWH dosage seems to be associated with lower incidence of mortality compared to standard DVT prophylaxys in hospitalized COVID-19 patients. Our study paves the way to further pathophysiological investigations and controlled studies of anticoagulation therapy in Covid-19 disease.

## Background

Recently, a novel coronavirus (2019-nCoV), officially known as severe acute respiratory syndrome coronavirus 2 (SARS-CoV-2), caused a severe pandemic infection worldwide with considerable morbidity and mortality. The name for the disease resulting from SARS-CoV-2 infection has also been identified with the acronym COVID -19 [Co (corona); Vi (virus); D (“disease”) and 19 (the year of virus identification)]. This is associated with an increase of systemic inflammation, cytokine storm and diffuse endothelial injury, resulting in the most severe cases in multi-organ failure with consequently poor outcomes. Fortunately, the clinical spectrum appears to be wide, including asymptomatic infection, mild upper respiratory tract illness and severe viral pneumonia with occurrence of respiratory failure (Zhou et al., 2020). Despite virus lung tropism using the angiotensin-converting enzyme 2 protein of alveolar cells, COVID-19 could induces cardiovascular complications too, including acute cardiac injury, ischemic heart disease, venous thromboembolism, myocarditis, heart failure and tachyarrhythmias (Driggin et al., 2020). The main clinical symptoms are fever, dry cough, fatique, ageusia and anosmia. So far, there is no specific therapy for COVID-19; antiviral therapy is recommended and chloroquine or hydroxychloroquine has been suggested as having antiviral activity. As the cytokine storm appears to be a crucial pathogenetic process in COVID-19 patients, immunosuppression and immune modulation approaches have been tried by glucocorticoids, monoclonal antibodies against IL-6R. Low molecular weight heparin (LMWH) administration in COVID-19 patients has been recommended by some expert consensus due to the risk of primary pulmonary thrombosis, venous thromboembolism and disseminated intravascular coagulation (World Health Organization; Driggin et al., 2020; Spyropoulos et al., 2020). However, evidence about administration dosage and duration is limited and its efficacy on clinically relevant endpoints being yet to be demonstrated.

To address such unsolved issues, we analyzed data from a single referral center to investigate the potential association between different dosages of LMWH enoxaparin administration and mortality among hospitalized COVID-19 patients.

## Methods

Consecutive patients admitted to Sant’Orsola Bologna University Hospital with laboratory-confirmed COVID-19 were included in this retrospective cohort study, which was conducted from March 01, 2020, to April 10, 2020. The diagnosis of COVID-19 was established according to World Health Organization interim guidance and confirmed by RNA detection of the SARS-CoV-2 in the microbiology laboratory of the hospital (World Health Organization).

**Figure 1:**
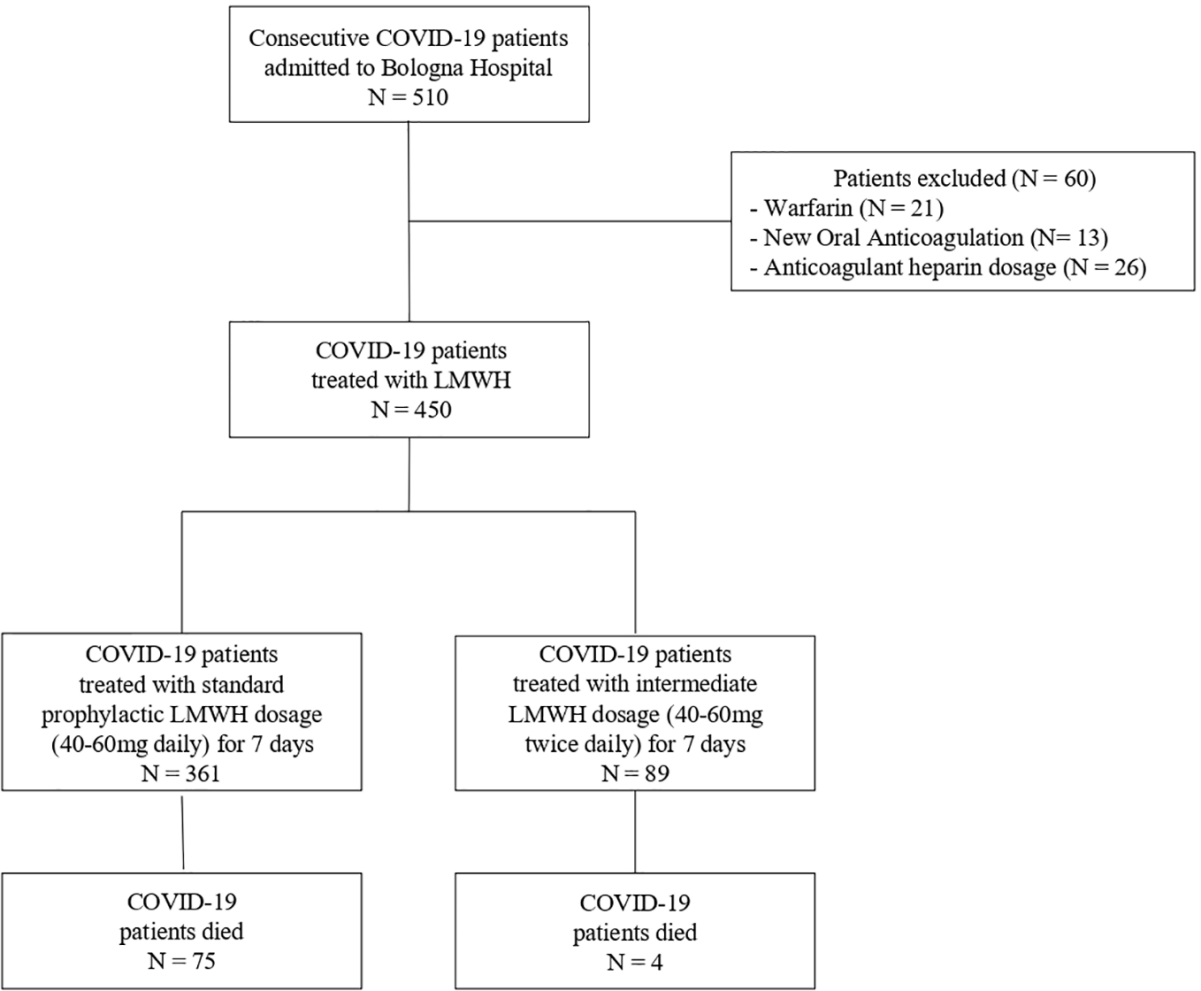
Flow chart study.

Outcomes between COVID-19 patients treated with standard prophylactic LMWH enoxaparin dosage (subcutaneous enoxaparin 40–60 mg daily) or intermediate LMWH enoxaparin dosage (subcutaneous enoxaparin 40–60 mg twice daily) for 7 days were compared (**Figure 1**).

The decision to adopt standard prophylactic or intermediate LMWH dosage was based on the clinical judgment of the attending physician.

Exclusion criteria were a bleeding diathesis, hospital stay < 5 days, lack of information about coagulation parameters and medications, age <18 years and any disease dictating anticoagulation, such as atrial fibrillation, prosthetic heart valves, or venous thromboembolic disease.

After discussion with infectious disease specialist and signature of informed consent by the patient, the majority of patients were treated with Tocilizumab (8 mg/kg - maximum: 800 mg/dose - every 12 hours for two doses) and/or hydroxychloroquine (400 mg twice daily on day 1, followed by 400 mg/day in two divided doses, for a total treatment duration of 5 days), when appropriated (Yao et al., 2020). Clinical, laboratory, radiological, and treatment data were collected and analyzed.

Outcome measurements included all-cause-death, severe respiratory failure requiring admission to the intensive care unit (ICU) and/or orotracheal intubation and/or renal failure requiring continuous venovenous hemofiltration (CVVH) and/or extracorporeal membrane oxygenation (ECMO), acute kidney injury (AKI), acute myocardial infarction, atrial fibrillation and heart failure.

Severe COVID‐19 was defined as meeting arterial oxygen saturation ≤93% at rest or PaO_2_/FiO_2_ ≤ 300 mm Hg. We did not include respiratory rate ≥30 breaths/min according to the Diagnosis and Treatment Plan of COVID‐19 suggested by National Health Commission of China due to the considerable inter-observed variability.

Non evidence-based treatments were administered according to DL 18, art.17/3/2020 of the National Health Service for the Emerging Infectious Disease. The need for informed consent was waived accordingly.

### Statistical Analysis

Continuous and categorical variables were presented as median (IQR) and n (%), respectively. We used the Mann-Whitney U test, χ. test, or Fisher’s exact test to compare the two groups of heparin dosage, as appropriate. To compare the all-cause in-hospital mortality between the two groups of heparin dosage, logistic regression analysis with propensity score adjustment was used, to control for the imbalance in the group characteristics. The propensity score, i.e. the conditional probability of being treated with the intermediate LMWH dosage given the set of variables that differed significantly between the dosage groups, was estimated using a multiple logistic regression model.

## Results

We evaluated clinical records of 510 consecutive adult patients with confirmed COVID-19 diagnosis referred to our Hospital. Twenty-one treated with warfarin, 13 patients with new oral anticoagulation and 26 patients taking anticoagulant enoxaparin dosage (100 mg twice daily) were excluded. A total of 450 hospitalized patients with COVID-19 infection were included in the final analysis (**Figure 1**). Three-hundred-sixty-one of them received standard prophylaxis LMWH treatment compared with 89 patients receiving intermediate LMWH dosage for 7 days. As reported in **Table 1**, no significant differences in the main demographic characteristics and laboratory testing at admission were observed in the two heparin regimen subgroups, except for older age and prevalence of hypertension in the group treated with “standard” prophylaxis LMWH dosage (p=0.048 and 0.01 respectively). A slightly worse degree of respiratory failure (PaO_2_/FIO_2_ value; p = 0.003) was observe in the group of standard prophylaxis LMWH, however the severity of COVID 19 was similar between the two groups. Patients treated by the intermediate LMWH dosage received hydroxychloroquine and i.v. Tocilizumab more frequently (p = 0.01 and p = 0.002, respectively). Despite a similar baseline profile, no differences in ICU admission, orotracheal intubation, renal failure requiring continuous venovenous hemofiltration and extracorporeal membrane oxygenation between the groups were observed. Interestingly, the intermediate LMWH dosage was associated with a lower in-hospital all-cause mortality compared to the standard prophylaxis LMWH dosage (18.8% vs. 5.8%, p = 0.02). This difference remained statically significant after adjustment with the propensity score for potential confounder variables that differed significantly between the dosage groups (age, hypertension, hemoglobin value, PaO_2_/FIO_2_ value, administration of hydroxychloroquine and Tocilizumab; OR= 0.260, 95% CI 0.089 - 0.758, p=0.014).

**Table 1:** Demographic, clinical, laboratory findings, treatment, and outcomes of COVID-19 patients at admission.

	Total N = 450	Standard Prophylactic LMWH dosage (40-60 mg daily) N = 361	Intermediate LMWH dosage (40-60 mg twice daily) N = 89	p-value
Age, years, median (IQR)	67 (55-79)	67 (55-79)	60 (54-74)	0.048
Sex, n (%)				0.9
Female Male	167 (37) 283 (63)	134 (37) 227 (63)	33 (37) 56 (63)	
BMI > 30 Kg/m^2^, median (IQR)	26 (24-30)	26 (24-29.7)	26 (24-29)	0.98
***Cardiovascular risk factors***				
Current/past smoking, n (%)	106 (23.6)	81 (22.4)	25 (28.1)	0.3
Hypertension, n (%)	228 (50.7)	194 (53.7)	34 (38.2)	0.01
Dyslipidemia, n (%)	53/212 (25)	37/155 (23.9)	16/57 (28.1)	0.5
Type-2 diabetes, n (%)	65 (14.4)	52 (14.4)	13 (14.6)	0.9
***Medical history***				
Previous AMI, n (%)	37 (8.2)	32 (8.9)	5 (5.6)	0.3
Chronic obstructive lung disease, n (%)	58 (12.9)	47 (13)	11 (12.4)	0.9
Chronic kidney disease, n (%)	30 (6.7)	25 (6.9)	5 (5.6)	0.6
Active cancer, n (%)	51 (11.3)	45 (12.5)	6 (6.7)	0.1
***Clinical presentation***				
Fever (temperature > 37.3 C°), n (%)	392 (87.1)	313 (86.7)	79 (88.7)	0.6
Cough, n (%)	271 (60.2)	203 (56.2)	68 (76.4)	0.006
Dyspnea, n (%)	169 (37.5)	129 (35.7)	40 (45)	0.2
HR, median (IQR)	81 (72-94)	80 (72-93)	81 (72-94)	0.9
SBP, median (IQR)	120 (110-135)	120 (110-135)	120 (120-130)	0.6
DBP, median (IQR)	70 (70-80)	70 (70-80)	75 (70-80)	0.2
***Laboratory parameters***				
Hemoglobin g/dl, median (IQR)	13.2 (11.7–14.6)	13.2 (11.8–14.6)	13.8 (12.3–14.8)	0.05
White blood cells N/µl, median (IQR)	6205 (4615–8865)	6140 (4455–8455)	5610 (4405–8640)	0.6
Lymphocyte %, median (IQR)	20 (13–28)	21 (15–29)	20 (13–28)	0.3
Platelet count x 10^9^ per L, median (IQR)	194 (151–256)	194 (148–252)	193 (154–248)	0.9
Blood glucose level mg/dl, median (IQR)	108 (95–136)	107 (94–131)	108 (98–131)	0.3
Creatinine, median (IQR)	0.91 (0.74–1.2)	0.92 (0.75–1.16)	0.87 (0.71–1.05)	0.08
C reactive protein mg/dl, median (IQR)	6.3 (2.2–11.4)	5.9 (2.2–11.5)	6.3 (2–11)	0.6
Alanine aminotransferase U/L, mean (SD)	26 (17–42)	27 (16–41)	28 (20–42)	0.5
Aspartate aminotransferase U/L, median (IQR)	34 (24–48)	34 (24–47)	34 (25–47)	0.9
Lactate dehydrogenase U/L, median (IQR)	293 (225–385)	287 (221–377)	288 (228–374)	0.9
Creatine kinase U/L, median (IQR)	84 (53–188)	91 (56–192)	78 (50–152)	0.3
Interleukin-6 pg/mL, median (IQR)	29 (12.6–64)	28.5 (13–64)	30 (12–68)	0.9
D-dimer μg/mL, median (IQR)	0.8 (0.5–1.5)	0.8 (0.5–1.6)	0.7 (0.5–1.2)	0.5
Procalcitonin ng/mL, median (IQR)	0.1 (0.1–0.5)	0.1 (0.1–0.4)	0.1 (0.1–0.4)	0.4
High sensitivity Troponin ng/L, median (IQR)	16.4 (7.6–59.4)	13.8 (7.6–47.5)	23 (5.9–96.5)	0.6
***Arterial Blood Gas***				
SpO2, median (IQR)	96 (94–98)	96 (94–98)	96 (94–98)	0.5
PaO2, median (IQR)	71 (60–82)	69 (60–80)	72 (65–85)	0.01
FiO2, median (IQR)	21 (21–28)	21 (21–28)	21 (21–25)	0.3
PaO2/FiO2 ratio, median (IQR)	310 (233–357)	305 (231–352)	329 (267–371)	0.003
SpO2 ≤ 93% or PaO2/FiO2 ratio ≤ 300, n (%)	202/389 (52)	162/306 (53)	40/83 (48.2)	0.44
***Therapy***				
Hydroxychloroquine, n (%)	363 (80.7)	283 (78.4)	80 (89.9)	0.01
Tocilizumab, n (%)	72 (16)	48 (13.3)	24 (27)	0.002
***Outcomes***				
All-cause Death, n (%)	79 (17.6)	75 (20.8)	4 (4.5)	0.001
ICU admission, n (%)	70 (15.6)	57 (15.8)	13 (14.6)	0.8
Orotracheal intubation, n (%)	62 (13.8)	53 (14.7)	9 (10.1)	0.2
CVVH, n (%)	6/237 (2.5)	5/180 (2.7)	1/57 (1.8)	0.7
ECMO, n (%)	4/237 (1.7)	3/180 (1.7)	1/57 (1.8)	0.4
AKI, n (%)	16/237 (6.8)	12/180 (6.7)	4/57 (7)	0.9
Stroke, n (%)	1/237 (0.4)	1/180 (0.56)	0 (0)	0.5
AMI, n (%)	5/237 (2.1)	4/180 (2.2)	1/57 (1.8)	0.8
Atrial fibrillation, n (%)	13/237 (5.5)	12/180 (6.7)	1/57 (1.8)	0.1
Heart Failure, n (%)	4/237 (1.7)	3/180 (1.7)	1/57 (1.8)	0.9
Hospital length of stay days, median (IQR)	10 (6–14)	10 (6–13)	8 (6–14)	0.4

Continuous variables are presented as median (IQR) while categorical ones as n (%) or n/N (%), where N is the total number of patients with available data.

AMI, acute myocardial infarction; BMI, body max index; PaO2, arterial partial pressure of oxygen; FiO2, fraction of inspired oxygen; HR, heart rate; SBP, systolic blood pressure; DBP, diastolic blood pressure; ICU, intensive care unit; CVVH, renal failure requiring continuous venovenous hemofiltration; ECMO, extracorporeal membrane oxygenation; AKI, acute kidney injury.

No fatal hemorrhages/bleeding occurred in either treatment group. Two major bleeding events (both gastro-intestinal bleedings) in the standard prophylaxis LMWH group and 2 (both retroperitoneal hematomas) in the intermediate LMWH dosage group occurred (0.6% vs. 2.2%, respectively; p = 0.13). Moreover, hemoglobin values remained stable after one week of therapy in the two groups analyzed (12.2 ± 2.1 vs. 12.4 ± 1.7 g/dl; p = 0.3).

## Discussion

Our data suggest that the standard prophylaxis LMWH dosage for DVT may be less effective than the intermediate LMWH dosage. In fact, in-hospital mortality rate is significant lower in our cohort of COVID-19 patients treated with intermediate LMWH dosage and this difference remained significant after adjustment with the propensity score for variables that differed significantly between the dosage groups.

Previous report suggested that standard prophylaxis LMWH dosage is associated with an improve outcomes in COVID-19 patients. Tang et al. demonstrated that LMWH treatment was associated with better prognosis in severe COVID‐19 patients meeting “Sepsis-Induced Coagulopathy” (SIC) criteria or with markedly elevated D‐dimer (Tang et al., 2020). However, this study compared heparin vs. no heparin strategy, a quite unusual procedure in obliged bed rest/bedridden hospitalized patients. Moreover, Tang et al. selected only severe patients affected by COVID-19 pneumonia. Conversly, we considered all patients without a clinical indication to anticoagulation therapy independently of severe and non severe COVID-19 and we compared two different LMWH strategies.

Recent guidance document recommends the use of standard-dose regimens with LMWH in the management of venous thromboembolism (VTE) in hospitalized COVID-19 patients, primarly in severe COVID-19 patients, although data on efficacy and safety of this approach are limited (Spyropoulos et al., 2020). So far, there are no studies that evaluate the efficacy, safety and duration of LMWH in non-severe COVID patients.

In the present article, we evaluated the different LMWH regim dose (“standard” versus intermediate LMWH enoxaparin dosage) in all hospidalized patients without stritifyng the severity of COVID-19 infection. The main novelty of our work is that for the first time we demonstrated that intermediate LMWH enoxaparin dosage improved the prognosis both in severe and non-severe COVID-19 patients. However, we do not examine the exact mechanism of this evidence. The beneficial effect of intermediate LMWH regimen observed in our study could be correlated to the prevention of or pulmonary microthrombosis and/or disseminated intravascular coagulation, which are considered as possible pathophysiological mechanisms leading to a worse prognosis in COVID-19 patients (Driggin et al., 2020; Llitjos et al., 2020; Poissy et al., 2020).

Although in this study we did not assess the thromboembolic complication, literature data demonstrated that massive pulmonary thrombosis/embolism was the cause of death until one third of the case (Deshpande, 2020; Klok et al., 2020). However, this data should be confirmed because of the limited number of autopsy studies available. Pulmonary thrombosis in COVID-19 probably develops as a consequence of vascular damage produced by virus infection and the resulting severe inflammation, with the pathogenic role of platelets and leukocytes, interacting with the vascular wall (Mcguire et al., 1982; Schattner, 2017). About this, it seems that heparin exerts an anti-inflammatory action and endothelial protection independently of its anticoagulant properties, however these ant-inflammatory and anti-thrombotic are potentially inter-related. The cytokines and particularly the interleukin (IL; IL- 1β, IL-6 and IL-8) are known to play an important role in inflammation and have direct effect on the plasma molecules, on erythrocytes and platelets, triggering an hypercoagulation state. Hypercoagulability and compromised fibrinolysis are usually the characteristics of numerous inflammatory disorders. In particular, patients with COVID-19 infection had higher levels of IL-6 which induced an inflammatory condition and aggravate the hypercoagulation status (Zhou et al., 2020; Huang et al., 2020; Wang et al., 2020). Moreover, heparin might help other drugs to inhibit the effects on COVID-19 infection through the direct antiviral effect, anticoagulation and the antinflammatory propretiers (Esko and Lindahl, 2001).

Our findings of significantly less in-hospital mortality rate in patients treated with intermediate LMWH dosage supported the hypothesis that LMWH accomplish simultaneous antithrombotic, anti-inflammatory and antiviral activity in COVID-19 infection. These effects seem to be more effective in intermediate LMWH dosage.

In our study, a significantly higher number of patients in the intermediate LMWH dosage were treated with both hydroxychloroquine and tocilizumab. Tocilizumab, which is the IL-6 receptor antagonist, has been approved for the treatment COVID-19 patients. Although, the administration and dosage of tocilizumab for COVID-19 are still being explored and the risk of serious infection (neutropenia or thrombocytopenia, and liver damage) must be investigate. In this patients the association of LMWH might help to reduce inflammatory burden and consequently the dosage of tocilizumab. Regarding the role of hydroxychlorochine, some observational studies have recently demostrated that administration of hydroxychloroquine did not reduce the risk of intubation or death in COVID-19 patients and its benefits remain very weak and conflicting (Geleris et al., 2020; Hernandez et al., 2020). So far, there is no clinical evidence that chloroquine/hydroxychloroquine is effective against Covid-19 and only large, randomized, placebo-controlled clinical trials will show if this drug is effective and safe to use as a treatment for Sars-CoV-2 infection.

However, there are substantial limitations of our observation owing to its retrospective and non-randomized nature, namely the absence of a formal hypothesis testing and some unbalanced features of the study subgoups.

In conclusion, although the optimal thromboprophylactic regimen is not known, our study opens new insights in heparin therapy for patients hospitalized with COVID-19. Large clinical trials are needed to define: a) a more precise dose-range of LMWH to balance the anti-thrombotic action and potentially severe bleeding complications; b) the more appropriate disease stage in which LMWH administration should be started; c) the clinical fingerprint of patients most likely to benefit from LMWH therapy.

## Data Availability Statement

The raw data supporting the conclusions of this article will be made available by the authors, without undue reservation, to any qualified researcher.

## Author Contributions

PP, LB, ECD, and MG contributed conception and design of the study. FD, ST, RP, MBa, and GT organized the database. LB and MBi performed the statistical analysis. PP wrote the first draft of the manuscript. PP, LB, and MG wrote sections of the manuscript. BC, PV, CP, MBi, and NG revised the article. All authors contributed to the article and approved the submitted version.

## Conflict of Interest

The authors declare that the research was conducted in the absence of any commercial or financial relationships that could be construed as a potential conflict of interest.

## Abbreviations

AKI, acute kidney injury; COVID -19, Corona Virus Disease 19; CVVH, continuous venovenous hemofiltration; DVT, deep vein thrombosis; ECMO, extracorporeal membrane oxygenation; ICU: intensive care unit, LMWH: low molecular weight heparin; SARS-CoV-2, severe acute respiratory syndrome coronavirus 2; VTE, venous thromboembolism.
